# Improving Data Glove Accuracy and Usability Using a Neural Network When Measuring Finger Joint Range of Motion

**DOI:** 10.3390/s22062228

**Published:** 2022-03-14

**Authors:** James Connolly, Joan Condell, Kevin Curran, Philip Gardiner

**Affiliations:** 1 Letterkenny Institute of Technology, F92 FC93 Letterkenny, Donegal, Ireland; 2 School of Computing, Engineering & Intelligent Systems, Ulster University, Londonderry BT48 7JL, UK; j.condell@ulster.ac.uk (J.C.); kj.curran@ulster.ac.uk (K.C.); pvgardiner@gmail.com (P.G.)

**Keywords:** data glove, sensor calibration, joint range of motion, kinematics, neural network

## Abstract

Data gloves capable of measuring finger joint kinematics can provide objective range of motion information useful for clinical hand assessment and rehabilitation. Data glove sensors are strategically placed over specific finger joints to detect movement of the wearers’ hand. The construction of the sensors used in a data glove, the number of sensors used, and their positioning on each finger joint are influenced by the intended use case. Although most glove sensors provide reasonably stable linear output, this stability is influenced externally by the physical structure of the data glove sensors, as well as the wearer’s hand size relative to the data glove, and the elastic nature of materials used in its construction. Data gloves typically require a complex calibration method before use. Calibration may not be possible when wearers have disabled hands or limited joint flexibility, and so limits those who can use a data glove within a clinical context. This paper examines and describes a unique approach to calibration and angular calculation using a neural network that improves data glove repeatability and accuracy measurements without the requirement for data glove calibration. Results demonstrate an overall improvement in data glove measurements. This is particularly relevant when the data glove is used with those who have limited joint mobility and cannot physically complete data glove calibration.

## 1. Introduction

Sensor data gloves have been successfully used in many use cases to detect finger joint movement of the hand including virtual reality (VR) [1], robot control [2], gesture recognition [3], animation modeling [4], medicine [5] and rehabilitation [6]. Mainstream data gloves typically use piezoresistive [7], fiber-optic [8], hall effect [9] or inertial measurement unit (IMU) [10] sensors to detect finger joint movement. Data gloves with divergent sensor constructions have demonstrated similar measurements within a series of accuracy and reliability tests [11,12,13,14,15,16], and proved their viability as an alternative goniometric measurement, with the added advantage of minimizing the common inter-tester and intra-tester reliability problem [17].

Data glove manufacturers design a data glove so that glove sensors are placed above each finger joint they wish to capture joint movement from. However, the human hand does not conform with a standard template. Finger, knuckle and palm length and circumference differ, as do range of motion (ROM) values between each finger joint, and between genders [18,19]. Typical flexion ROM at metacarpophalangeal (MCP) and proximal interphalangeal (PIP) finger joints increase linearly from the index to little finger by up to 13 degrees [5,13,20]. Additionally, goniometric studies focusing on the effects of age on ROM [21] found a correlation between age and decreasing joint flexion. Furthermore, women generally have greater hyper-extension flexibility for all finger joints than men [20]. 

Therefore, data gloves are designed with compromise in mind to match the average human finger and hand length and circumference to average small, medium, or large hand templates. Data glove compromises can have a detrimental effect on data glove fit, which in turn affects accuracy and reliability. Sensors within a data glove can be misaligned with their associated finger joints if the wearer’s hand is slightly dissimilar to the glove’s sensor layout design. A data glove cloth “skin” supports, constrains, and protects data glove sensors and circuitry from the environment. However, the multi-layered design can also have a detrimental effect on glove accuracy and reliability. Typically, a two-layered construction is implemented on a data glove skin; the inner layer provides sensor and electronics positioning and support, while the outer layer protects sensors and electronics from the environment. Glove sensors are positioned within the inner and outer cloth layers.

Data glove manufacturers typically require completion of a sensor calibration process before each use that aims to reduce the effects of poor skin fit or sensor misalignment. Software provided with a data glove usually controls each step of the calibration process. Typically, a calibration routine requests the user to position each group of finger joints in specific poses as advised by this software. For example, the “flat hand” position captures MCP and PIP minimum sensor readings, the “fist” position captures maximum values for MCP and PIP sensors, and the “thumb touching palm” position captures maximum thumb flexion measurement. Sensor values captured from each sensor at each calibration step are used to set the minimum and maximum ROM sensor settings for each finger joint for that specific data glove wearer. The calibration process is not compulsory, but it is an important initialization step needed to achieve maximum sensor accuracy. The process is time consuming, and data glove accuracy can still be quite inadequate. Research by Huenerfauth and Lu [22] commented on the poor quality of the accompanying software calibration process for their Cyberglove III data glove. They discussed how the complex calibration processes typically lasted for 75–90 min to calibrate one glove for one user. They devised an advanced calibration process to improve overall accuracy by implementing an individual finger calibration routine that included gain and offset values for each glove sensor.

Data gloves have the capability of objectively measuring various disease characteristics such as joint stiffness caused by rheumatoid arthritis [23], but complex calibration can lessen the ability to measure such important conditions since inaccurate calibration significantly reduces data glove accuracy. Data glove calibration may not be possible when wearers have disabled hands or limited joint flexibility, such as those suffering with osteoarthritis, post-stroke complications, or Parkinson’s disease. If a glove wearer is incapable of providing flexion and extension movement during calibration, precision greatly diminishes for each glove sensor and the data glove becomes unusable for the wearer. Accurately determining the minimum and maximum ROM for a glove wearer with inadequate flexion and extension capabilities becomes difficult without using external hardware such as a goniometer. Accurate angular calculation therefore relies on calibration measurements that are close to the normal ROM values that are typical ROM movement for a human hand.

The application of an intermediary method between data glove sensors and their calculated movement and resultant output could improve glove accuracy and repeatability when glove fit is a problem and remove the need for calibration for those with limited joint mobility. A few classification methods have been used in conjunction with data gloves to bypass the need for calibration when used for grasp recognition, and gesture recognition/pattern recognition [19]. Heumer et al. [24] used various neural networks (NN) to analyze uncalibrated sensor data from their data glove when the wearer grasped various objects. They discovered that uncalibrated data could be used with a classifier for reliable object recognition for repeatedly grasped objects. Zhou et al. [25] implemented a bespoke calibration method in conjunction with a camera to measure each finger joint and select the most suitable gain and offset values to use as calibration settings for each sensor of their Cyberglove III data glove. They trained NNs with gain and offset data and then used camera images to select the most suitable calibration settings. Their research concluded with positive detection of five postures. Their research removed the need to complete the calibration process, although a complex camera setup was required to implement their alternative calibration method. Kahlesz et al. [18] concluded it was possible to use a data glove without the need for calibration using out-of-the-box NN classifiers. They examined the cross-coupling effect between MCP joints of their Cyberglove III data glove [26] and the adjoining abduction/adduction sensors. They used lookup tables for fast evaluation of abduction angles generated by the data glove sensors. Kahol et al. [27] used a Hidden Markov Model (HMM) to recognize electroencephalogram (EEG) data combined with Vicon [28] movements for sign language gestures.

The motivation of this paper is to evaluate whether it is possible to implement a NN to circumvent the data glove calibration process and improve overall accuracy and reliability of a data glove. If successful, this technique could be adopted for data glove use when data glove sensor non-linearity is an issue, or when data glove calibration is not possible due to limitations of finger joint mobility. This method could include those with limited joint mobility, for whom data glove calibration and use are not possible or are very limited.

## 2. Materials and Methods

To demonstrate the benefits of implementing the NN method for data glove calibration, accuracy and repeatability were assessed for an off-the-shelf data glove using testing techniques devised from previous data glove research. Although no formal set of data glove accuracy and repeatability testing strategies exist, the methods originally devised by [16] and later adapted by [13,29,30] were modified for this study. The data glove was firstly assessed for accuracy and repeatability using the default 5DT data glove calibration process provided with 5DT’s proprietary software. Then, a NN was constructed for each data glove sensor, and accuracy and repeatability testing were applied to the NN method. Finally, both methods were statistically compared to determine whether a NN improved data glove accuracy and repeatability.

### 2.1. Data Glove Used in This Study

**Figure 1:**
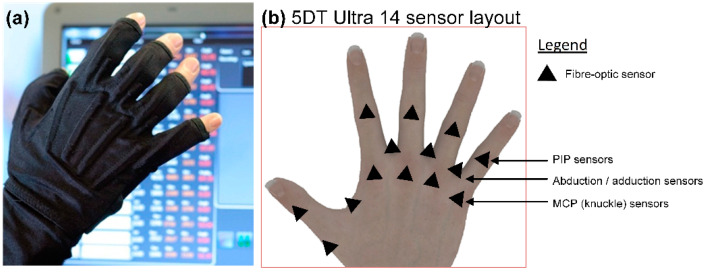
(**a**) 5DT data glove with bespoke controlling software in image background. (**b**) Diagram showing sensor positioning for the 5DT data glove.

The 5DT Data Glove 14 Ultra shown in Figure 1a was selected for this study. It is produced to fit medium hand size 7 ½ to 8 [31]. 

It uses stretchable Lycra to support its 14 fiber-optic sensors that are placed over the MCP, PIP joints, and between each finger to measure abduction/adduction of the MCP joints as shown in Figure 1b. Joint movement is detected by the LED–phototransistor sensor construction as a change in the intensity of light passed along each sensor’s fiber-optic loop. Each glove sensor generates 12 bits of data that describe the position of each sensor using an integer output within the range of 0–4095. For this study, bespoke controlling software was devised and written by the research team to manage data glove functionality, including calibration, NN construction and control, and angular measurement and recording for each data glove sensor.

### 2.2. Neural Network Architecture

Feed-forward back-propagation (BP) and radial basis feed-forward (RBF) are the most commonly used NNs for pattern recognition [32]. A BPNN is an algorithm whose learning is based on the deepest-descent technique, and is constructed of two parts [33]. The feedforward propagation part of the NN accepts an input that is propagated into hidden units within each layer of the NN until a result is produced [34]. Then, the back propagation part examines the error rate calculated from the current iteration (epoch) and tunes each weighted value between each layer to decrease the error rate and improve the overall accuracy of the BPNN. As this part of the process implies, the errors and the learning rate “propagate” backwards from the output nodes to the inner nodes of the network. The goal of the back propagation aspect of the network training is to optimize each interconnecting “weight” so that the network can correctly map arbitrary inputs to outputs. The BPNN carries out the requested epochs and attempts to achieve a state where outputs are similar to desired outputs. After a specified number of epochs, the BPNN should have selected the most adequate weights that are closest to the desired output. 

**Figure 2:**
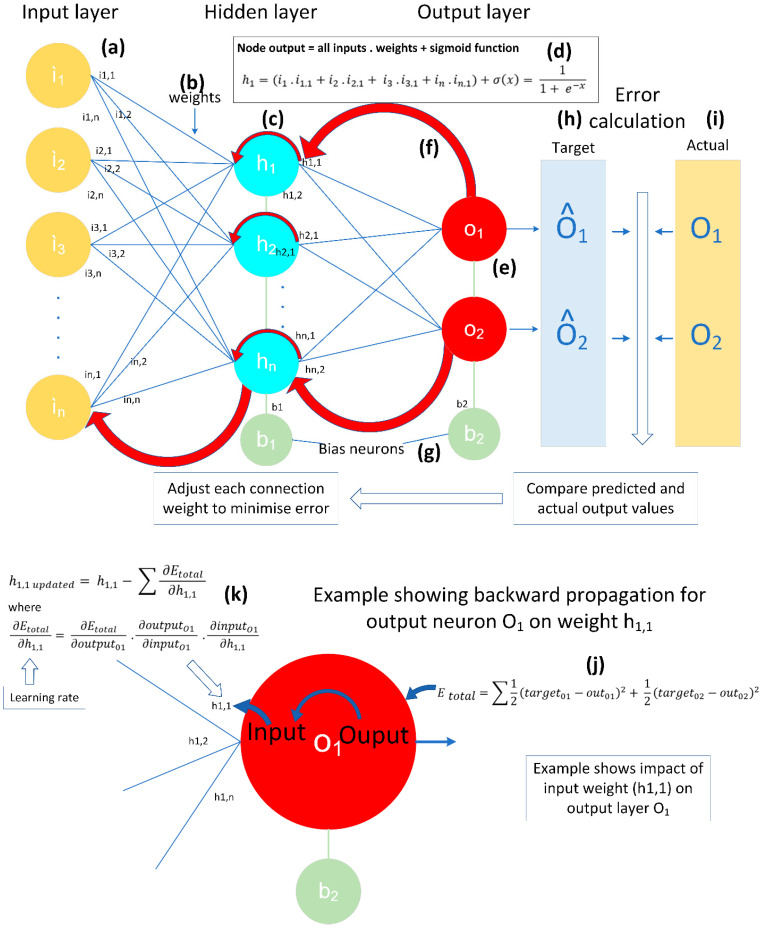
Top diagram shows the number of input, hidden and output layers implemented in the BPNN used in this study. The red arrow signifies the back propagation stage of the training process. Network inputs (**a**) consist of data glove sensor readings, (**b**) shows weights between each node and layer which are adjusted by the back propagation process as training progresses. (**c**) shows the hidden layer nodes which are carefully considered to avoid underfitting and overfitting. (**d**) demonstrates the mathematical function used to sum each layer’s inputs and calculate its sigmoid activation function. The backward propagation process then begins (**f**). The bias node is initialized, but is not influenced by inputs from previous layers (**g**). The output layer (**e**) compares the preferred pattern (**h**) with the hidden node outputs (**i**). Bottom diagram shows an example of the process needed to generate the total error for the current epoch (**j**) and the process to create a new weighed value for input h1,1 (**k**) using the sum of all inputs combined with a sigmoid function.

A BPNN is constructed of three “layers”, as illustrated in Figure 2a,c,e (top diagram). Each “node” in each layer is connected to every other node in that same layer through interconnecting weights, and each layer is connected to the other layers through interconnecting weights between nodes in other layers. The input layer (Figure 2a) is influenced by the number of features within the data. In this study, the number of input nodes was set at 13 for BPNNs built to represent MCP joints, and 14 for BPNNs constructed for PIP joints. The number of hidden layers (Figure 2c) was carefully considered to avoid “underfitting” when too few neurons are available to correctly model a complex dataset, and “overfitting” when not enough information is available to train all the nodes within the hidden layer. As a general rule of thumb, the number of hidden neurons should be a value between the size of the input and output layers. In this study, 7 nodes were selected for the hidden layer. The number of output nodes in the output layer (Figure 2f) was chosen based on the output requirements of the NN. In this study, two outputs were required: one to represent each sensor value output, and one for the corresponding angular value.

The BPNN was trained in several stages. Firstly, some small random values were assigned to each of the weights between each node (Figure 2b) and the bias node (Figure 2g). In a BPNN, the bias node does not receive an input from a previous layer. Instead, the bias update is determined by a change in the gradient of the total error (Figure 2j). Then, the feedforward part of the training process was initiated whereby each input node received an input value from a data frame which stored recorded pairs of sensor and angular data from the data glove. In this study, the middle PIP glove sensor was initially used for experimentation to build the logic around the BPNN implementation due to its close fit for all hand sizes of study participants. 21 sample points were extracted for the middle PIP finger joint between 0° and 105° in 5° angular increments. The data from each input node were transmitted to each connected node within the hidden layer. Each hidden layer node (Figure 2c) summed its input values, calculated its sigmoid activation function (Figure 2d), and then sent its signal to each node in the output layer (Figure 2e). The mathematical process for the sigmoid activation function is shown in Figure 2d for the hidden node “h1”. The sigmoid activation function ensured that linearity did not influence any of the calculations within the BPNN. The output layer completed the same processes as the input layer. Each of the output layers also received a target pattern which represented the target values expected from the BPNN (Figure 2h). The initial output values were not expected to be close to the actual values (Figure 2i) due to the initial randomization of the weighed values for each node’s interconnections.

The overall error for the BPNN for that epoch is then calculated as the total error for all output nodes. This is the commencement of the backward propagation part of the training process (Figure 2f), and the mathematical process is shown in Figure 2j. The gradient, also known as the partial error, is calculated for each weight within the BPNN, as shown in Figure 2k, and they are summed to produce the total adjusted weights for each layer. Finally, all of the weights are updated and the process is repeated for the number of epochs defined at the initialization of the BPNN.

**Figure 3:**
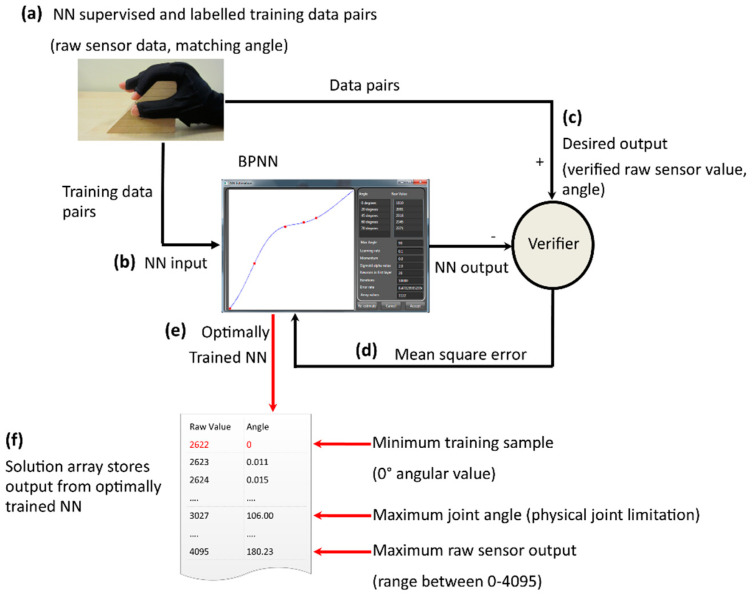
Training raw input data (**a**) and angular inputs (**b**) provide predicted training sample points for NN training. During its training phase, the output of each NN epoch is compared with the desired output of each training sample (**c**). Mean square error is an indicator of agreement between input training samples and NN estimated values for each NN epoch (**d**). Once an NN is accepted (**e**), each iteration in a pre-created solution array is input to the NN. Each output of the NN is stored in the corresponding angle element in the array (**f**). Smoothing algorithm removes unneeded data pairs above the physical limitation of each glove sensor, shown here as the maximum joint angle. Each raw value is normalized using the minimum training sample value.

Figure 3 shows a high-level overview of the steps required to create and implement each of the PBNN calibrations. Data glove sensor information was extracted for each sensor from the data glove. Additionally, a BPNN was constructed for each sensor for 4 hand sizes (7, 7½, 7¾, 8 inch). Therefore, 10 BPNNs were generated for each hand size. Creating BPNNs for each hand size in small increments should minimize variances in users hand sizes and should improve overall angular accuracy compared to traditional linear calculations. The raw sensor information for each sensor, combined with the associated angular values were firstly input to the BPNN as predicted and labelled sample points of training data (Figure 2a and Figure 3a). Raw values were used for training input data, and angular inputs were used as training targets for the input data (Figure 3b).

The resultant NN model was then assigned to the relevant model representing the specific hand size that the model was trained for. The first set of BPNN models were configured for the 7¾ inch hand size. LR and momentum NN values were set at 0.1 to ensure optimum NNs were not overlooked, and each NN training session was run for 100,000 iterations (epochs). One training sample point (BPNN input layer) was removed sequentially from each NN training session to determine the best combination of training sample point inputs that achieved the lowest NN MSE. The number of BPNN inputs used in this study were examined to ensure that the optimum number of training sample points were chosen that reduced the incidence of local minima. Experiments found that optimum training sets using 13 training sample points for MCP glove sensors and 14 samples points for PIP sensors were best. Each sample point contained one raw sensor input data value and one angular value that represents the training target for input data. The input angles used were 0°, 5°, 15°, 25°, 30°, 35°, 40°, 50°, 55°, 65°, 75°, 85° and 95° for MCP finger joints, and 105° was additionally used with PIP glove sensors to account for typical greater flexion of this finger joint. NN testing was performed in the real-world using angles 0°, 20°, 45°, 60°, 70° and 90°. Angle 0° was used as a sample point and in real-world testing since this value sets the minimum angle for each sensor during training and is an important component of angular resolution from glove data.

Learning rate (LR) is a constant that influences step size taken along the gradient vector of the NN error surface. Larger steps may bypass a definitive NN and smaller steps require larger number of epochs. Momentum encourages movement in a static direction. If several steps follow the same path, the NN can compute values more quickly. Momentum can help escape local minima and encourage the NN to quickly jump over flat spots, although jumping may miss an optimum NN. Optimum LR and momentum values are determined through experimentation.

A two-dimensional solution array as demonstrated in Figure 3f held raw values and corresponding angular readings that were generated by the optimum BPNN created for each data glove sensor. Raw values in the solution array were created once the solution array was initialized and before NN training began. Angular readings were then predicted by the NN once training was complete. The lowest raw sensor value represented the start of the raw range and was the first value stored in the two-dimensional solution array. This lowest raw value was calculated from sensor output when the data glove wearer placed their hand on a flat surface. In Figure 3f, the first raw value for demonstration purposes is shown as 2622. This number represented the numerical value produced by the data glove sensor when placed on a flat surface. Once the lowest raw value was recorded and placed into the array, additional raw values were then calculated incrementally in single raw value steps up to the maximum integer value that could be generated by each glove sensor. For the 5DT data glove, the maximum raw sensor value was 4095. The error of each BPNN epoch was computed as the MSE of difference between training input data and those generated by the NN as shown in Figure 3d.

Once the number of requested epochs was reached, the NN epoch with the lowest MSE was selected. If an NN epoch was accepted as the preferred iteration, each raw value segment from the solution array was input into the NN and its output was then predicted by the optimum BPNN for each data glove sensor, as shown in Figure 3e. The solution array was examined by a “smoothing” algorithm devised to remove any unnecessary values beyond the physical limitations for that corresponding finger joint. Smoothing was applied after the full angular range was created by the NN. For example, the index MCP finger joint can flex on average to 95°. Angular values generated beyond this were discarded since they were not physically achievable by a glove wearer. Finally, the raw segment of each data pair in the solution array was normalized by subtracting the smallest raw value from each BPNN-generated value. The raw values were then used as a lookup key during angular calculation.

### 2.3. Implementing Neural Network Angular Calculation

**Figure 4:**
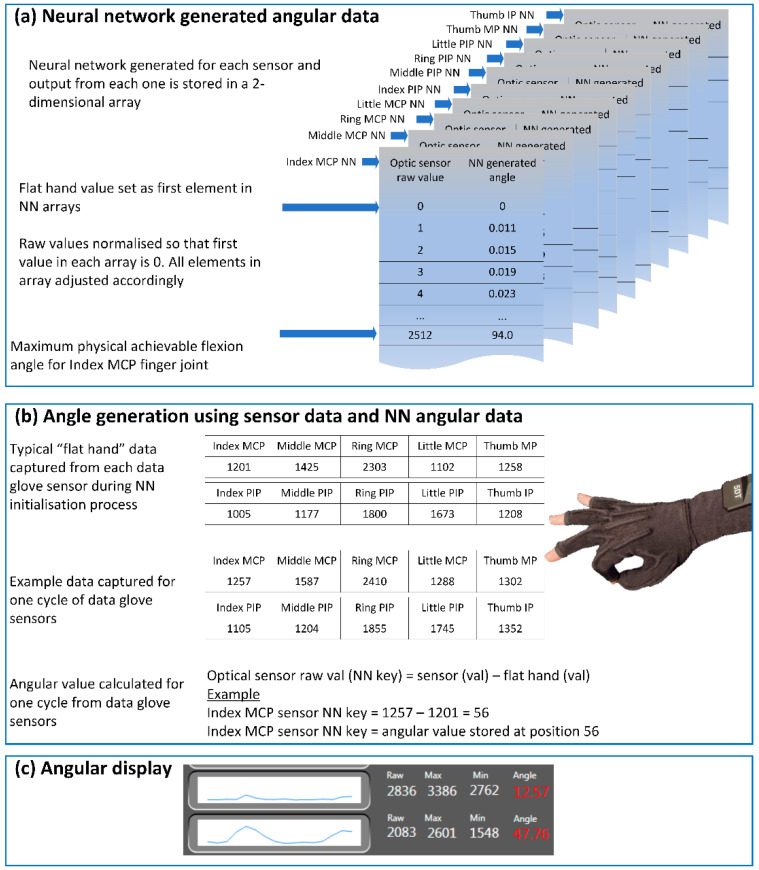
NNs were created for each data glove sensor. Optimum NN for each sensor was used to predict angular values for that sensor. (**a**) Predicted values for each sensor were stored in a 2D array and used for angular calculation. (**b**) Each angle was indexed by subtracting the “flat hand” value for each sensor from the sensor output. Index key indicates array position containing the correct angular reading for a sensors bend angle. (**c**) Angular readings were then displayed using controlling software.

Once the NN-generated angular values were constructed, they were used as lookup tables for angular calculations for each data glove sensor. The data glove-controlling software imported NN-generated angular ranges into a dictionary for each sensor. Angular calculations were not produced linearly from each data glove sensor; instead, angular values were generated and calculated using the raw sensor data from each sensor as the lookup key to locate the correct output angle for each data glove angle. Figure 4 presents the processes for NN angular calculation for each data glove sensor using the NN-generated angular value lookup tables. The optimum NNs were stored in an individual array for each sensor.

Ten arrays were used to represent each of the ten sensors in the 5DT data glove as shown in Figure 4a. Angular output for each sensor was generated using real-time raw sensor data that was subtracted from the stored “flat-hand” value for each sensor to create the lookup key (Figure 4b). Each lookup key contained the location within the relevant sensor array to the angular value as calculated by the BPNN. The angular value was then displayed within the data glove-controlling software (Figure 4c). In the example shown in Figure 4b, the “flat hand” value for the index MCP sensor was represented by the sensor value 1201. This was the numerical value that was output by the data glove index MCP sensor when the wearer placed their hand on a flat surface. This value was used to generate the lookup key for the 2D array. The example shown in Figure 4b shows that the index MCP sensor outputted a value of 1257 during one cycle of sensor output. Consequently, the lookup key for the index MCP sensor was 1257 − 1201 = 56 for that sensor output. Therefore, the angular value representing sensor value 1257 was contained in the indexed position in the MCP array that was stored in location 56.

### 2.4. Examination of the Benefits of Neural Network Calibration

As previously described, the data glove was firstly assessed for accuracy and repeatability using the default 5DT data glove calibration process provided through 5DT’s proprietary software. Then, the NN technique was implemented and both methods were statistically compared and analyzed to determine if the NN calibration method improved data glove accuracy and repeatability compared with traditional calibration. 

### 2.5. Repeatability and Accuracy Testing Strategies

Data were collected from four right-handed subjects, three male and one female. All males had hand sizes between 7½ and 8 inch (medium) and the female hand size was 7 inch (small). Wise et al. [16] found a significant difference between repeatability among male and female subjects. Dipietro et al. [12] subdivided their results into two groups due to the high level of difference between male and female results. Both research studies deduced the problems were caused by the anthropometric differences in hand sizes and ill fitting of the glove onto a smaller hand size.

The data glove was tested for repeatability and accuracy of measurement. Testing strategies previously proposed by [12,16,30] were applied to the data glove when it was calibrated using the standard calibration method and angular values were calculated using a standard mathematical formula. In this study, this method is referred to as the “linear” method since data were collected from each sensor when assuming linear stability across the full range of movement. The data glove was then calibrated using the NN calibration method, and angular values were calculated using lookup tables generated by a NN for each sensor. This method was referred to as the “NN” method.

Although a standard protocol does not exist for data glove testing, using the strategies devised by previous research will allow for comparison of results to earlier work. Tests A to C examined the ability of the data glove to consistently measure the same sample multiple times with the data glove. Test D examined the distance in degrees between the average value of each measurement and the true verified measurement from a goniometer.

In each test, readings were captured from the data glove using the research teams controlling software. Readings were averaged due to the high number of samples generated from the 50 Hz glove refresh rate (50 samples per second) for each MCP and PIP groups of sensors for each subject. Each group included index, middle, ring and little finger joint data. Data were captured for each test using the traditional calibration method.

### 2.6. Repeatability Testing

**Test A**: Plaster mold, glove on between measurements, angular values generated using glove sensors and NNs

In this test, each subject wore the data glove on their right hand. They placed their right hand with the data glove on a flat surface and positioned their forearm in a prone-supine position and with their right wrist in a neutral position. A template of a hand was drawn to ensure each subject positioned their right hand in the same flat surface location during each test cycle A test cycle for this test is when each subject placed their hand within the template for five seconds and then rested the palm of their hand for five seconds on the plaster mold. Each subject rested the palm of their hand on the plaster mold to reduce the effect of grip pressure on data glove readings. Each tests cycle was repeated 10 times without removing the glove. The same test was then repeated using the NN calibration method whereby angular data were collected using data generated from NNs.

**Test B:** Hand flat and then in a fist. Angular values generated using glove sensors and NNs

This test examined whether the material properties of the data glove had any effect on data glove sensor readings when the data glove was stretched and then positioned in the “flat hand” position. Each subject placed their right wrist and hand flat on a tabletop within the boundaries of a template with their wrist in a neutral position and forearm pronated. Each subject closed their hand to form a fist whilst wearing the data glove and then returned it to the flat position, within the template boundaries. In this test, a test cycle is when each subject completed one flat and fist clenched phase. Each test cycle was repeated 10 times within a set of tests, and each test was repeated twice. Angular values were initially calculated using data collected from each data glove sensor. The same tests were then repeated using angular data generated from NNs. Angular values were captured from each sensor when the data glove was in the “flat hand” position.

**Test C:** Hand flat and glove off between data collection

This test was conducted in the same manner as test B, except the data glove was removed between each test cycle. Additionally, the data glove was not recalibrated between each donning and doffing. This test simulated the effect of using one calibration method for multiple uses of the data glove. An initial calibration routine was completed when the data glove was first placed on the subject’s right hand. The same set of tests were then rerun using data generated from NNs.

### 2.7. Accuracy Testing

Finger joint ROM is routinely assessed in the clinical setting using a goniometer, and this is the current state-of the-art method for finger joint mobility assessment. A data glove needs to provide similar accuracy to goniometric measurement if it is to be used within a clinical environment to measure finger joint flexibility. Accuracy testing therefore examined and compared the data gloves ability to accurately measure finger joint angle and to compare with a goniometer. The accuracy testing strategy proposed by Williams et al. [30] was amended to determine if data glove angular readings were comparable to traditional measurement techniques. Additional tests were included to examine data glove capacity to measure maximum ROM.

**Test D:** Data glove on, generating angular values linearly and using NN

In this test, each subject wore the data glove on their right hand and sequentially placed each finger joint on top of wooden blocks with angles 0°, 20°, 45°, 60°, 70°, and 90°. The controlling software generated angular values for each finger joint when placed on each wooden block, and the average angular reading was recorded for each wooden block. The test was then repeated using the NNs to generate angular readings for each data glove sensor. Once these measurements were captured, the data glove was then removed, and a goniometer was used to measure finger joint angle for each finger joint when it was placed on top of each wooden block. A goniometer was placed over each finger joint and angular readings were captured from the goniometer. Angular readings using the linear and NN methods were then compared with the goniometric measurement.

**Test E:** Data glove on between maximum flexion routines

Each subject placed their hand on a flat surface with the forearm in a prone-supine position. Each subject completed two routines; a MCP flexion routine followed by a PIP flexion routine. Each routine required the glove wearer to maximally flex the relevant group of joints 12 times. Speed was unimportant, although each subject was requested to focus on moving just one set of joints in each test. Mean maximum ROM measurements were examined for each joint group. Angular readings were generated linearly first, and then using the NN-generated values. Previous studies by [20,35,36,37] indicate that mean maximum flexion for MCP joints is between 93 and 101 degrees, and between 110 and 114 degrees for PIP joints.

## 3. Results

Subject 1 reported the glove fitted loosely across the palm of the hand and over the little finger and thumb. Anthropometric variation of the small female hand to the medium-sized glove meant each sensor was not ideally placed over the corresponding finger joint. Each glove fingertip is open, allowing larger fingers to fit the medium glove frame. Subject 4 reported tight fitting of the glove across the palm of the hand and in each finger. Donning and doffing were quite difficult.

### 3.1. Repeatability Testing

Table 1 presents repeatability results for test A for each subject whilst collecting angular values from the data glove using the traditional angular calculation method. Table 2 presents the same repeatability results for test A when using NN-generated angular calculations. Since standard deviation (SD) is a measure of the amount of dispersion of a set of values from the mean, a low SD indicated that the angular values were typically close to the mean (expected value) and showed high levels of repeatability. Although overall average and SD values were similar between both methods of angular calculation indicating a close representation across measures to the expected mean value, individual subject results revealed significant variations. Subject 1 (S1) and subject 4 (S4) showed greater improvement when using NN-generated angular calculation compared to those generated linearly. Ill-fitting of the glove on S1 and S4 caused greatest variations in results when using the traditional angular calculation method. Using NNs to calculate angular values decreased this variation. Variations between S2 and S3 were minimal, indicating comparable repeatability accuracy between NN-generated angles and those generated linearly due to good fitting of the glove to both subjects’ hands. Both methods of angular calculation showed greatest variation in thumb IP joint accuracy. The molds may not have sufficiently stabilized the thumb position during recording, although NN repeatability values reduced the overall range variation for the thumb joints.

Table 3 presents the range of measurements between each of the test cycles for test B and test C and were analyzed from angular values recorded when the data glove was in the “flat hand” position. Results for test B indicated that the data glove had less range of “flat hand” values when using the NN method compared to the linear method. Additionally, the average range was less for the NN method compared to the linear method, indicating that there was greater stability and repeatability achieved from the NN method over the linear method.

Results from test C showed that angles generated linearly were minimally affected by the glove donning and doffing procedure, which suggested that a set of data glove calibrations are sufficient when using a data glove. NN angular accuracy was more greatly affected by data glove donning and doffing. The NN strategy relies more heavily on an initial accurate “flat hand” value to generate all subsequent angular readings than the linear method, suggesting that the NN method requires the “flat hand” process to be completed each time the data glove is placed on the wearer’s hand. Results consistently show less range for the NN angular calculation method than the linear generated method, and SD results also indicate that results using the NN method are more stable than the linear method.

### 3.2. Accuracy Testing

The results for test D are demonstrated as Pearson’s correlation coefficients (R^2^) that are used to compare the measure of linear correlation between measurements generated from the linear and NN methods with traditional goniometric measurement. Table 4 presents the R^2^ results of angles generated linearly using the data glove and compared with a goniometer. Results confirmed a high degree of association between angular readings calculated with the data glove and compared to the goniometer. The lowest correlation occurred for the MCP joints for S4.

R^2^ linear correlation levels for the NN method when compared with the goniometric measurements for test D are shown in Table 5. Results indicated an improved correlation using the NN method over the linear method, suggesting a higher level of linear correlation with goniometric measurement and NN-generated angular values compared with the linear method

**Figure 5:**
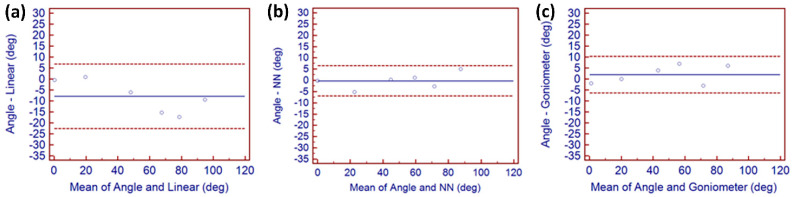
Bland–Altman limit of agreement charts for little MCP showing (**a**) levels of agreement between precise angles and those linearly generated by the data glove linear method, (**b**) levels of agreement of precise angles and those generated using NN technique, and (**c**) levels of agreement between precise angles and those recorded from a goniometer.

Examining the correlation between the expected true angle, goniometer measurements, and both data glove measurement approaches is essential to discover which method closely matched the expected true angular readings for test D and a Bland–Altman limit of agreement chart is used to display these relationships between both measurements. This type of chart is based on the quantification of the agreement between two quantitative measurements by studying the mean difference and then constructing limits of agreement by using the mean and the standard deviation of the differences between both measurements. It can be used to demonstrate whether, and how strongly, pairs of variables are related [38]. Dotted lines on each chart represents ± 2SD, and the middle blue line represents the mean difference between both measurements. Dotted lines close together represent small differences in SD and a good relationship between both variables. The Bland–Altman charts shown in Figure 5 represent limits of agreement for the little MCP finger joint. This joint was chosen because it was the most ill-fitting finger of the data glove and it represented the maximum differences between each method of measurement using the data glove. 

Figure 5a displays the limits of agreement between expected precise angles and those directly generated by the data glove using linear calculations. Highest levels of agreement were achieved by angles generated using the NN technique as shown in Figure 5b and were marginally better than those generated from the goniometer shown in Figure 5c, although the improvement provided by the NN method is also evident when compared with goniometric techniques. The level of agreement displayed in Figure 5c is comparable with agreement reported by other research work [12,16]. The reasonably horizontal spread of points across Figure 5b indicated that NN angular differences did not increase as values for the goniometer and data glove increased. The linear chart in Figure 5a does not show similar spread, indicating higher levels of change between the goniometer and data glove across the range of measurement. Variances between both measurement techniques are small for all other finger joint measurements.

**Figure 6:**
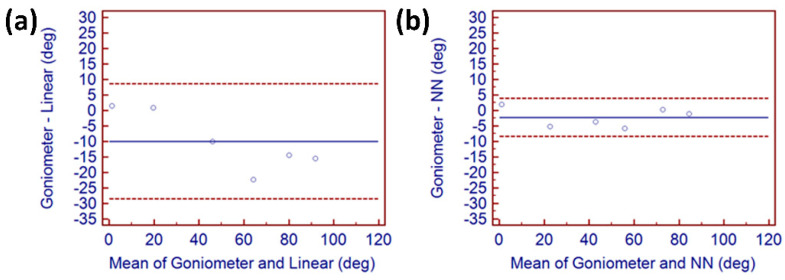
Bland–Altman agreement technique for little MCP. (**a**) demonstrates level of agreement between goniometer and angles generated from glove data. (**b**) demonstrates agreement between goniometer and NN angular calculation.

Since the goniometer is the currently accepted method used for finger joint assessment in a clinical environment, it was compared with measurements captured simultaneously with a goniometer and using the linear and NN data glove collection methods. Figure 6 demonstrates the level of agreement between a goniometer and angles generated by the glove and those generated by the NN technique. The improvement in accuracy when using the NN technique compared with the linear method is quite apparent. The uneven spread of points above and below the mean line (blue) in Figure 6a shows an uneven spread of points across all readings, indicating unpredictable angular measurement throughout movement. Accuracy was improved for minimum (0 degrees) and maximum (80 degrees) angles, and those values affect both flat hand tests and maximum flexion tests. These values were initially calculated during the calibration routine. Accuracy was mostly affected between 40 degrees and 80 degrees, which may have been caused by ill fitting of the glove to the wearer, or pressure on the underside of each sensor due to variances in finger thickness.

Figure 6b displays the agreement of NN-generated values to accurate angles. Improvements in angular accuracy compared to goniometric and linear angular values are immediately apparent. Readings remained consistently close to the mean angular blue line, indicating a strong agreement between the goniometric measurements and those generated using a NN. Results also indicated that angular values calculated linearly and those generated using the NN-generated range are both comparable to traditional goniometric measurement.

Results for test E are presented in Table 6 and Table 7. Both tables present the mean difference values and 95 percent confidence intervals of MCP (Table 6) and PIP (Table 7) sensor groupings for each subject, recorded using linear and NN angular generation methods. As described earlier, MCP joints generally have a mean maximum flexibility between 93 and 101 degrees, and PIP joints between 110 and 114 degrees. Therefore, results were expected to be within these general boundaries for each joint grouping. The mean maximum ROM difference shown in both tables represents the mean difference between all measurements and their distance from the 95% confidence boundary, therefore providing a general indication of measurement stability. A low mean difference score indicated that the results were reliably within the same ROM each time the participant completed the maximum ROM flexion. Results for subject 1 (small hand) show improvement, particularly for the PIP joint group, although an average mean difference of −11.3 degrees indicates a reasonably high level of inaccuracy still exists. Results for subject 4 (large hand) shown in Table 6 demonstrated a significant improvement using the NN method compared to linear generated glove values for MCP and PIP movement. The mean value for MCP maximum ROM (80 degrees) is closer to the mean expected value (93–101 degrees), compared to maximum ROM (127.8 degrees, mean difference of 37.86 degrees) with the linear calculation method. Similar improvements for subject 4 are shown in Table 6, since the mean maximum ROM (116.14 degrees) is closer to the mean for this group of joints (110–114 degrees) compared with 91.82 degrees maximum ROM and −18.2 degrees mean difference, although the mean difference in NN values indicates a high level of inaccuracy still exists (average −9.63 degrees MCP and 6.14 degrees PIP) for this hand size. In both subjects, ill fitting of the data glove caused the problem, and using the NN technique increased accuracy levels. Subjects 2 and 3 (medium hand) showed improvement for both groups of joint, and particularly PIP joints. Both subjects had medium hand sizes.

Accuracy was slightly improved for subject 1 using the NN method instead of the linear one. Subject 1 had a small hand size. Subject 4 had a large hand size and although maximum angular values generated using the NN method improved compared to accuracy with linearly generated values, overall accuracy was less than the expected maximum ROM for MCP joints. Table 7 presents improved results for the NN-generated angular values compared to those generated linearly.

## 4. Discussion

Using a data glove to replace traditional goniometric measurement devices has been investigated for a number of years by research groups. The gloves used in previous work were similar in material structure and sensor placement to the data glove used in this study, although the type of sensors used to detect joint movement varied between gloves used. All previous work discussed limitations of goniometric measurement tools currently used for the assessment of functional ROM for finger joint movement in a clinical environment. Using a semi-automated measurement process such as a data glove for hand assessment has the potential to reduce the time demand for both clinician and Occupational Therapist (OT). A data glove also offers simultaneous recording of each finger joint during the execution of tasks. 

A disadvantage of using a data glove is the requirement for calibration before each use. This may limit its usability in a clinical context if the data glove is to measure limitations in joint mobility and the wearer cannot flex and extend their finger joints to complete the calibration process. This study examined whether an alternative glove calibration and subsequent angular calculation process could be used to circumvent calibration and therefore open data glove use to those with limited joint mobility. The assessment techniques used in this study uncovered that both repeatability and accuracy measurements were improved for each test for each data glove user when a NN was used for calibration and angular generation from each data glove sensor. Furthermore, since a calibration process was not needed, this opened the possibility of those who could use the data glove to include those with limited joint mobility. Repeatability and accuracy recorded during this study are compared to results from previous studies and presented in Table 8.

As shown in Table 8, the 5DT glove used in this study compares repeatability to previous work. Results have been improved using NN-generated angular ranges to quantify real-time angular measurement compared to linear angular calculation from the 5DT glove. Test results have shown the glove used in this study can consistently provide repeatable angular readings. Using an NN-generated angular range improves results still further. When using the data derived from the glove using standard angular calculations from each sensor, its performance is most suited to fields where accuracy throughout the full movement range is not vitally important such as VR and gesture-based applications. NN-generated ROM for each data glove sensor provides the greatest advantage when angular readings vary throughout the full spectrum of movement of a sensor. Results in this study showed improvement when a subject wore the data glove that did not accurately fit their hand. The NN method removes the non-linear nature of movement for each sensor. This characteristic could increase overall movement accuracy for sensors that have a trait of nonlinearity, such as with bend sensors.

## 5. Conclusions

Connected health care and associated technologies are capable of monitoring various vital signs from the user and can assist the clinician to remotely monitor patient progression and patient diagnosis. Data glove hardware has the potential to be used for remote monitoring of disease progression of finger joint limitations and their improvement through rehabilitation. However, data glove calibration limits their use to those with normal ROM. This study identified shortcomings in data glove calibration and configuration methods and proposed a novel method to overcome these issues. It is envisaged that data glove manufacturers could use this method to construct a set of NN calibration lookup tables that bypass the requirement for complex calibration, and instead automatically calibrate the data glove to suit the hand size of the wearer. 

## Figures and Tables

**Table 1:** Repeatability results for test A. Angular readings were generated using data collected from each data glove sensor.

	MCP				PIP				Thumb (Mean)		Mean
	S1	S2	S3	S4	S1	S2	S3	S4	MCP	IP	
Range	2.25	0.78	1.61	1.25	1.18	0.61	1.28	0.84	1.05	3.13	1.69
SD	0.64	0.19	0.43	0.26	0.25	0.11	0.27	0.18	0.21	0.73	0.28

S1: subject 1; S2: subject 2; S3: subject 3; S4: subject 4. SD: standard deviation. Range represents the variance from minimum to maximum recorded angular readings for joint groupings for each subject. SD shows the average dispersion of values within each range.

**Table 2:** Repeatability results for test A. Angular readings were generated using NN lookup tables for each data glove sensor.

	MCP				PIP				Thumb (Mean)		Mean
	S1	S2	S3	S4	S1	S2	S3	S4	MCP	IP	
Range	0.89	0.98	1.50	0.85	1.10	1.17	0.99	0.69	0.57	2.05	1.07
SD	0.19	0.18	0.34	0.20	0.26	0.27	0.25	0.13	0.12	0.45	0.23

S1: subject 1; S2: subject 2; S3: subject 3; S4: subject 4. SD: standard deviation. Range represents the variance from minimum to maximum recorded angular readings for joint groupings for each subject. SD shows the average dispersion of values within each range.

**Table 3:** Repeatability results for test B and test C.

Subject	Test B Range (SD)		Test C Range (SD)	
	Linear	NN	Linear	NN
S1	12.43 (3.42)	10.36 (3.58)	8.67 (3.44)	11.84 (3.44)
S2	4.25 (1.26)	3.97 (1.33)	6.67 (1.97)	3.15 (0.96)
S3	5.57 (1.69)	4.23 (1.33)	6.78 (1.92)	7.92 (2.31)
S4	6.72 (2.46)	4.98 (2.01)	6.51 (1.93)	3.10 (1.04)
Average	7.24 (2.20)	5.88 (2.06)	7.15 (2.31)	6.50 (4.40)

**Table 4:** Pearson’s correlation showing comparison of goniometric values versus angles generated linearly from data glove.

	MCP Joint				PIP Joint			
Digit	S1	S2	S3	S4	S1	S2	S3	S4
I	0.996	0.990	0.989	0.991	0.990	0.985	0.895	0.996
M	0.827	0.971	0.978	0.924	0.992	0.954	0.975	0.811
R	0.986	0.950	0.980	0.898	0.999	0.997	0.990	0.918
L	0.979	0.993	0.940	0.877	0.971	0.948	0.956	0.985
T	0.960	0.975	0.948	0.997	0.913	0.978	0.850	0.831

Pearson’s correlation coefficient of angular values calculated for test D. S1: subject 1; S2: subject 2; S3: subject 3; S4: subject 4. I: index; M: middle; R: ring; L: little; T: thumb. Correlation showing level of agreement of angles captured with goniometer and those captured linearly. Results segmented per finger joint group.

**Table 5:** Pearson’s correlation showing comparison of goniometric values versus NN-generated angles from data glove.

	MCP Joint				PIP Joint			
Digit	S1	S2	S3	S4	S1	S2	S3	S4
I	0.994	0.991	0.997	0.997	0.991	0.995	0.991	0.979
M	0.927	0.997	0.991	0.915	0.986	0.977	0.977	0.877
R	0.986	0.999	0.991	0.909	0.981	0.996	0.992	0.946
L	0.992	0.987	0.989	0.842	0.966	0.960	0.960	0.986
T	0.961	0.978	0.993	0.993	0.914	0.995	0.991	0.875

Pearson’s correlation coefficient of angular values calculated for test D. S1: subject 1; S2: subject 2; S3: subject 3; S4: subject 4. I: index; M: middle; R: ring; L: little; T: thumb. Correlation showing level of agreement of angles captured with goniometer and those captured using NN method. Results segmented per finger joint group.

**Table 6:** Average NN and linear comparisons for MCP joints.

Subject	MCP NN (Degrees)				MCP Linear (Degrees)			
	Mean	Mean Diff	95% Confidence Interval of Difference		Mean	Mean Diff	95% Confidence Interval of Difference	
			Lower	Upper			Lower	Upper
**1**	88.9	−0.08	82.03	90.86	86.86	−3.13	85.39	90.86
**2**	90.9	0.99	90.13	92.12	89.68	−0.31	84.94	93.30
**3**	90.3	0.25	89.08	91.59	92.71	2.71	91.13	94.29
**4**	80	−9.63	75.7	83.47	42.8	−17.86	32.61	43.10

**Table 7:** Average NN and linear comparisons for PIP joints.

Subject	PIP NN (Degrees)				PIP Linear (Degrees)			
	Mean	Mean Diff	95% Confidence Interval of Difference		Mean	Mean Diff	95% Confidence Interval of Difference	
			Lower	Upper			Lower	Upper
**1**	98.62	−11.3	94.2	98.54	80.67	−29.3	79.28	82.67
**2**	111.27	1.27	110.1	112.4	104.82	−5.17	98.79	104.5
**3**	104.36	−5.63	101.4	106.8	99.28	−9.84	94.32	108.1
**4**	116.14	6.14	112.4	119.8	91.82	−18.2	84.33	92.1

**Table 8:** Comparison of ‘flat hand’ and plaster mold test results from previous data glove research and results from this study. Range values represent the average range of measurements calculated for participants for each test within each study. SD represents the average standard deviation values for each test.

Study	Flat Hand Test (Range/SD)	Plaster Mold Flat Hand Test (Range/SD)
[16]	4.4 (2.2)	6.5 (2.6)
[12]	3.84 (1.23)	7.47 (2.44)
[39]	1.49 (0.5)	5.22 (1.61)
[40]	2.61 (0.86)	6.09 (1.94)
This study (linear method)	2.7 (0.995)	7.19 (2.11)
This study (NN method)	1.39 (0.33)	6.19 (3.23)

## Data Availability

The data presented in this study are available on request from the corresponding author. The data are not publicly available due to ethical reasons.
